# BRAIN HEALTH IN COMMUNITY: INVOLVING AND COLLABORATING WITH COMMUNITIES YIELDED UNEXPECTED INNOVATIONS

**DOI:** 10.1093/geroni/igae098.2018

**Published:** 2024-12-31

**Authors:** Daniel Gan, Claire Wang, Susan Liu Woronko

**Affiliations:** University of Toronto, Toronto, Ontario, Canada; Johns Hopkins University, Baltimore, Maryland, United States; Frog Hollow Neighbourhood House, Vancouver, British Columbia, Canada

## Abstract

Funded by university-community engagement initiatives, this research aimed to understand and meet gaps in cognitive health promotion in diverse communities. The research team started with CONSULTATIVE conversations with activity providers and partnered older adults for analytic model specification and interpretation. As the focus groups progressed, the engagement fluidly progressed into an INVOLVEMENT marked by mutual respect for the distinct expertise of both parties and quick successions of knowledge exchanges, which led to two unintended innovations. Due to the trust built with key informants, older adults openly shared their unmet needs for a space to find meaning amid the challenges of aging. In response, a novel mindful discussion program was COLLABORATIVELY piloted with community organizations. Moreover, trust from older adults begot trust from activity providers and their sharing led to a simple eMental Health solution for closed-loop social prescribing. These insights on trust, engagement levels, and innovations are gained only in retrospect. Referencing these experiences, we reflect on the importance trust built with older adults as a catalyst for deeper engagements with service providers. Low-intensity consultations were more manageable at the start of the researcher-community relationship. More extensive engagements with older adults led to greater trust and mutual empowerment, and incidentally provided access to implicit knowledge held by activity providers which was crucial for innovation. Pivotal moments of knowledge spillovers were part of everyday activities in the community as researchers became embedded in relationality. Our experience underscores fluid engagement approaches that defy our best-planned intentions as lessons in decolonial knowledge cultivation.

